# Retention of Urine

**Published:** 1858-08

**Authors:** Wm. B. Alley


ART. III.—*Retention of Urine.* An Address read before the Allegany Co. Medical Society. By WM. B. ALLEY, M. D.

MR. PRESIDENT AND GENTLEMEN:

It was resolved at our last meeting, that each member report some case occurring in his practice, with such practical suggestions as he may deem proper to make.

In compliance with this rule, I have taken the liberty to make some general remarks on vesical retention of urine, partially illustrating its causes, symptoms and treatment, with such cases as have come under my immediate observation.

Retention of urine, its causes and effects, are not as well understood, I fear, by many of us country physicians, as many maladies occurring less frequently. It is often met with under circumstances which render it a very troublesome and alarming malady both to physician and patient.

The symptoms of retention of urine in the bladder, are the existence in the hypogastric region of a hard pyriform, well defined tumor, more or less tender on pressure, and gradually increasing in size; if retention be combined with incontinence of urine, the distention goes on gradually increasing until the bladder, in some instances, acquires enormous dimensions, and has occasionally been mistaken by able men for other complaints. In the beginning, there may or may not be pain and a frequent desire to void urine; but commonly there soon comes on an urgent desire to pass water, which if passed at all, comes away in drops or small quantities; these symptoms being soon followed by pain, straining, tenesmus, and finally, by extreme restlessness and indescribable suffering. If the diagnosis is doubtful, catheterism will generally remove all doubts.

The causes of retention of urine are numerous, rendering the treatment frequently difficult and uncertain, unless we fully comprehend the particular cause in each case.

The direct or immediate causes, for convenience, may be divided into two classes:

*First:* into such as occasion in some manner a loss of the expulsive power of the bladder, viz., compression of the brain, paralysis, large anodyne doses, inflammation, over distention, blows, lacerated wounds, capital operations, fevers, and the habit of resisting the desire to urinate.

The *second* class are such as obstruct or result in obstructing the neck of the bladder or urethra, mechanically or otherwise, viz., calculi in the neck of the bladder or urethra, stricture, disease of the prostate gland, tumors and abcesses of the bladder or neighboring parts, inordinate distention of the rectum, injuries during parturition, hernia, coagulated blood spasms from the use of irritating drugs, and retroversion and prolapsus of the uterus.

I will now give short reports of a few such cases as have occurred from the first class of causes:

July 1st, 1846. Mrs. T. Cossett, of North Almond, aged 68, was taken with a rush of blood to the head, which resulted in apoplexy, hemiplegia of the left side and retention of urine, requiring the daily use of the catheter until death, which occurred six days afterward.

March 8th, 1849. Philoman Gilbert, of Hornellsville, aged 24 years, had fever, ending in typhoid form of seventeen days' standing, with retention of urine of two days' standing.

Catheterism twice a day, stimulating liniments and frictions applied to the spine and abdomen, and the internal use of muriated tincture of iron continued ten days, restored to the bladder its expulsive power.

This case I supposed to have been induced by the fever.

During the winter of 1848, Mrs. Enos, of Almond, aged 28, had dismenorrhœa, for which she took large doses of laudanum; two days after taking one of these doses, I found her restless, in pain, frequently trying to void urine, with a circumscribed tumor in the hypogastric region, as large as a child's head. Between three and four pints of urine were drawn by the catheter. Cold applications and catheterism twice more, effected a cure in thirty-six hours.

This must have been a case of temporary paralysis, produced by the laudanum, for two months afterward, I again treated her for retention of urine occurring under like circumstances.

June 14, 1849. Mrs. S. Withey, of Hornellsville, aged about 23, was in labor and had retention of urine; three pints of urine were drawn off, and after six hours sickness she was delivered of her second child. Twenty-four hours after, I found her still unable to urinate. There were no inflammatory symptoms. The vagina and external organs of generation were not much sore or swollen.

This was a case of temporary inertia or paralysis of the bladder, induced by compression from the uterus and fœtus, before and during labor. It required catheterism for fourteen days, and was cured by the use of general tonics and the application on the eleventh day of a large blister to the hypogastrium.

August 20, 1854. I saw Bartlet Dake, of Almond, aged 81 years, who had a large circumscribed tumor in the hypogastrium, which had been gradually increasing in size for more than two weeks. Mr. D. had been injured about the hips two months before, by a fall; since then he has had a kind of general marasmus, with more or less pain in the lower back and perineum. Three weeks ago he began to lose the control of his water, and for the last ten days there has been incessant dribbling of urine; he has considerable pain and tenderness in the bowels, and some pain in the perineum, with an occasional desire to urinate. Dr. Robertson, his physician (and a very respectable one) up to about this date had regarded it a case of incontinence of urine with inflammation of the bladder. When we proposed to introduce the catheter, the patient demurred, saying look at his wet bed and cloths, and that he knew there was no water in the bladder. I found it difficult to introduce the catheter, and after two or three attempts the patient again demurred, begging me to desist and declaring the urine had all come away, and if I would do something to remove the bloat in the bowels and ease his pain, he would soon be better. I gave him half grain of morphine. Four hours after I found the urethra less irritable, when with a different catheter I drew off about nine pints of urine. This case required catheterism until death, which occurred about four weeks afterward.

Aug. 27th, 1857. Joseph Platt, of Angelica, aged 49, was taken with general paralysis. Sixteen hours afterwards, in the absence of Dr. Charles, his physician, I found him in great pain, with an urgent desire to urinate, amounting to almost spasms from retention of urine. Introduced the catheter and evacuated about three pints of urine. Colchicum and various remedies were given, frictions and liniments continued externally for fifteen days, but the power to urinate had not returned, although he had begun to flex and extend one leg; and to move his arms in some directions.

Sept. 22d. Arnica, and blisters, and galvanism, had been used, still he required the frequent use of the catheter. Galvanism and stimulating embrocations were continued, and he took $\frac{1}{16}$ of a grain of strychnine, $\frac{1}{8}$ of a grain of cantharides, and 3 grains of extract of arnica, three times a-day. I do not know but Dr. Charles gave some other remedies.

October 1st, he had regained the ability to urinate, and about this time he began with assistance to walk, and has never had any return of retention of urine since; he has continued to improve in general health and strength, although there is partial paralysis of his hands and left leg remaining.

Jan. 4th, 1858. Miss Mary Blair, of Angelica, aged 13 years, died of hydrocephalus. Five days previous to death, she had retention of urine, which required catheterism twice a-day.

Jan. 4th. John Collins, aged about 85, was taken dangerously ill with typhoid pneumonia. On the 23d of January I saw him in consultation with Dr. E. M. Alba; found he had complete paralysis of the bladder, producing retention of the urine. There was quite a large tumor in the hypogastrium, yet the patient had no pain or desire to urinate. The doctor had used the catheter two or three times before. Mr. Collins being delirious, insisted that the introduction of the catheter was uncalled for, and submitted to its use with great reluctance. I had difficulty in introducing the instrument, from enlargement of the prostate gland; this, however, had no agency in causing the retention, but occasioned considerable trouble in introducing the catheter, until the doctor became acquainted with his particular deformity and had a catheter adjusted to suit.

This case required catheterism for about three weeks longer. He took buchu, muriated tincture of iron and general tonics. As soon as his muscular system generally became strong, the ability to urinate returned.

I will now give a few cases that have occurred from the second class of causes:

In March, 1848, T. M. Gary, aged about 20, had what I diagnosed as inflammation of the neck of the bladder and urethra following gonorrhœa, and terminating in suppuration. He had throbbing pain in the perineum; scalding sensation in the urethra; straining and a constant desire to urinate, followed in two days by retention of urine, attended with rigors, violent straining and the most excruciating pain. After making several unsuccessful attempts to introduce such catheters as I happened to have, I had Dr. C. D. Robinson called, requesting him to bring his catheters, who came, and by using more force than I had used, reached the bladder with one of his instruments. Several ounces of pus flowed at first, followed by a quart or more of urine mixed more or less with pus. The retention ceased, requiring no further treatment.

Oct. 27th, 1854. After thirty hours severe labor, Mrs. Clark Tifft was confined with her first child. Two days after, I found her with a high fever and a hard tumor in the supra-pubic region. The vagina and the external organs of generation, were much swollen and painful. She had not urinated since her delivery. The mentus urinarius was so tender and so much displaced by the swelling, that I could not introduce the catheter without an ocular view of the parts, and then the urethra was so extremely irritable that catheterism brought on spasms the first and second time.

Rigid antiphlogistic treatment, a dose of asafœtida, camphor and morphine given an hour and a half previous to the use of the catheter, accompanied with the application of cloths, wrung out of warm water, to the vulva, prevented a recurrence of the spasms. This thus far was a case of retention from pressure and injury during parturition, resulting in swelling and inflammation of the urethra and external organs of generation. The fever, swelling and tenderness, all subsided in ten days, when I found that she had complete paralysis of the bladder, as she could go thirty-six hours without any desire to urinate. This condition I have no doubt was the result of allowing the bladder to become over-distended. There appeared to be a general relaxed condition of the muscular system. Instead of once in thirty-six hours we used the catheter twice a-day; tonics and a generous diet was tried for one week; her general health improved, but the inability to urinate continued. A large blister over the hypogastrium was added to the treatment, and after three days more, I had her take in addition, one ounce of the infusion of buchu and $\frac{1}{20}$ of a grain of strychnine twice a-day, and had the urine drawn off three and four times a-day; in six days more I dismissed the patient cured.

Aug. 20th, 1854. Mrs. Wygaut, of Almond, a healthy woman, aged about 25. Ten days after confinement, had a chill, followed by fever and severe pain in the region of the uterus. I then diagnosed the case to be inflammation of the uterus, but the next day she had retention of urine. Catheterism caused considerable pain, which increased at each introduction of the catheter. On the fifth day the nervous system became greatly unstrung, the pulse frequent (130 in a minute); she was restless and in agonizing pain. On introducing the catheter, pus flowed at first instead of urine. The pain and bad symptoms soon abated. There was more or less pus discharged with the urine for a few days. The power to urinate returned on the second day after the first discharge of pus, accompanied with considerable pain for a few days. The pus had an extremely offensive smell; in two or three days the patient recovered her usual health in all respects.

About the 20th of August, 1857, Ezra Starr, aged 75, while on a visit at Bloomfield, New Jersey, had inflammation of the bladder, which left him with paralysis of that viscus and consequently retention of urine, requiring catheterism daily. On the 10th of Sept. his general health had sufficiently improved to enable him to start for home. Dr. Davis, his physician, gave him the name of some physician in Binghampton, and of Dr. Purdy, at Elmira, to call upon, if catheterism became necessary. He got into the cars at New York, and owing to some delays, and perhaps the peculiar motion of the cars, an urgent desire to urinate came on, compelling him to stop at about the first village, to have his water drawn off. As soon as he got to a hotel, he sent for a physician, who came and after making several unsuccessful attempts to introduce a catheter, called in another physician, and after hurting him terribly, and trying several times, they succeeded in breaking a silver catheter, but not in getting it into the bladder. These manipulations occasioned such indescribable suffering that the old gentleman begged them desist, feeling, as he says, that they did not understand their business, for Dr. Davis had introduced the catheter once and twice a-day for several days before, without the slightest difficulty; but they told him he would surely die unless his urine was drawn soon. He then allowed the third doctor to be called, who came, and they all tried and failed again, when Mr. Starr again asked them to desist; but they insisted that the urine must be evacuated at once, and attempted to make farther trials by holding him by main force, when the old gentleman commanded them to leave him. On the next morning he got another physician who, without very great difficulty, introduced the catheter, and emptied the bladder. During the same day Mr. Starr came on to Elmira, where Dr. Purdy also used the catheter without serious trouble; and on the 12th he came home to Angelica. I was soon asked to see him, the messenger saying that he was anxious to know "if any physician here could use a catheter in his case?" I found him feeble, feverish and in great pain, with an urgent desire to urinate. I succeeded, on the second trial, in getting the catheter into the bladder. I think the retention at this period was caused by the swelling of the urethra in part, but mainly by the clots of blood.

Sept. 13th. I found that the urethra had been ruptured in two places. Just as the catheter arrived at about the opening in the triangular ligament, it turned to the right, and when introduced about an inch, it would go no further. Next time when I got near the prostate gland, it suddenly turned to the left; its withdrawal was followed by considerable hæmorrhage. It became necessary to hold the instrument in a certain direction, and at each time exactly alike, in order to run clear of these artificial openings and reach the bladder.

On the 16th, an hour after catheterism, I was called in haste to relieve his urgent desire to urinate, when half a pint of blood was discharged by the catheter. I was called twice more during the day, when blood and urine were discharged. He complained of considerable pain in the back and loins.

On the 17th I catheterized him four times, large quantities of blood were discharged with the urine, obstructing the catheter so much that I was obliged sometimes to withdraw it and re-introduce it several times before all could be discharged.

Tonics, anodynes and styptics had been given, and cold applications applied to the perineum. Still the bleeding continued, and the feebleness increased.

Sept. 18th. About as yesterday, only catheterism was attended with excruciating pain, and followed by almost spasms. To-day Dr. Charles was called to assist in determining whether any part of this blood came from the kidneys, &c. Dr. Alba also saw him. We concluded that the blood came from the bladder, and the injury connected with it.

Sept. 19th. He was worse.

Sept. 20th. In the morning the clotted blood filled the catheter and nothing was discharged. At noon blood and urine were discharged.

Sept. 21st. He was some better, the urine was mixed with dark colored blood; appetite improved.

22d. The dark blood and urine continued as yesterday.

26th. Had a return of hæmorrhage.

27th. Perhaps a pint of blood was discharged.

Oct. 1st. The urine again dark, being mixed with blood.

10th. General health much improved. The urine became nearly natural except there was occasionally some blood mixed with it, or found at the bottom of the vessel.

12th. Strength and appetite improving, but catheterism was followed by severe pain, lasting two or three hours, requiring the use of anodynes.

20th. Was able to ride out.

Nov. 1st. Began to void urine in drops, and the pain after catheterism had subsided.

6th. Catheterized him for the last time; since then he has always been able to urinate without help, but sometimes he has been obliged to walk across his room a few times before the water would start.

Dec. 27th, 1854. I saw at North Almond, a male child of James Crausons, two days old. It had never urinated; upon examination I found the glans penis slightly enlarged, and no external opening to be found. Dr. E. W. Robertson, who was present, also examined but found none. The glans penis appeared all solid, there was no urethra there; I thought I could feel the bladder above and behind the pubis; also that I could feel the urethra back or between the scrotum and perineum. I then pushed a narrow bistoury one-third of an inch in the direction of the urethra, withdrew it and introduced a whalebone probe, but it would not go any further than I had cut. I then cut down about half an inch further, again tried the probe and found it passed beyond where I had introduced the bistoury and into the urethra. I removed the probe and introduced a catheter, when the urine flowed freely. We dressed it with a well oiled round tent, to be withdrawn and replaced once in eight hours. On the 10th of January, 1855, the child was well. I saw it again in June, 1857, the child was sound in all respects.

Gentlemen, I will not trouble you longer with the recital of cases. I have given, I trust, a sufficient number to satisfy all, especially our younger medical friends, that retention of urine occurs quite often and from a variety ot causes.

Retention of urine in the bladder of old people, indicates, when not brought on by injuries or obstructions, that the vital energies of the system are failing and is generally followed by death in a few months; but aside from this it is curable in the main.

Having already occupied so much of your time I do not feel at liberty to comment upon the treatment of this malady, and will only add a few words upon part of it.

All, I believe, concede that the treatment must be commenced by the introduction of the catheter. To fail in this little operation might prove a sad thing for our reputation, while to succeed will afford us some pleasure and our patient great ease and happiness. But this, like all other operations, requires an intimate acquaintance with the anatomy and position of all the parts connected with it both in disease and health; and no one who values his reputation or cherishes a proper regard for human suffering and life, will attempt it without these pre-requisites; our own interest in general, and that of our patients in particular, demand that we should well understand the rules and modes of introducing a catheter under all circumstances.

I will close by giving a short quotation from Prof. S. D. Gross upon this subject:

"The introduction of the catheter, although apparently very simple, is one of the nicest and most delicate processes in surgery. It requires skill of the highest order, as well the most intimate knowledge of the anatomy of the urinary organs. If I were called upon to state what I considered as the most important operation that a practitioner is obliged to perform, I should unhesitatingly say the introduction of the catheter. It is true, the most untutored and awkward surgeon may occasionally, nay, perhaps not unfrequently reach the bladder without difficulty; but let such an individual attempt the passage of the instrument when there is some mechanical obstacle, as a stricture or an enlarged prostate, and he will sure to be foiled. The moment the catheter is arrested he becomes bewildered, his hand trembles, dismay is depicted in every feature, large drops of sweat stand upon his brow, and his whole frame is paralyzed. If, under these circumstances, he proceed, he will inflict severe suffering upon his patient, if not actually endanger his life. To avoid such an occurrence, as disgraceful as it is unfortunate, the operation should be constantly practised upon the dead subject; the anatomy of the urinary apparatus should be thoroughly studied; and the eye, hand and instrument should be trained to move in concert with each other."

